# Anti-Diabetic Medications for the Pharmacologic Management of NAFLD

**DOI:** 10.3390/diseases6040093

**Published:** 2018-10-03

**Authors:** Rosann Cholankeril, Vikram Patel, Brandon J. Perumpail, Eric R. Yoo, Umair Iqbal, Sandy Sallam, Neha D. Shah, Waiyee Kwong, Donghee Kim, Aijaz Ahmed

**Affiliations:** 1 Department of Medicine, Roger Williams Medical Center, Providence, RI 02908, USA; rtc1088@gmail.com; 2 Charles E. Schmidt College of Medicine, Florida Atlantic University, Boca Raton, FL 33431, USA; vikrump@gmail.com; 3 Department of Medicine, Drexel University College of Medicine, Philadelphia, PA 19129, USA; brandonperumpail@gmail.com; 4 Department of Medicine, Santa Clara Valley Medical Center, San Jose, CA 95128, USA; eric.r.yoo@gmail.com; 5 Department of Medicine, Mary Imogene Bassett Hospital, Cooperstown, NY 13326, USA; umairiqbal_dmc@hotmail.com; 6 Division of Gastroenterology and Hepatology, Stanford University School of Medicine, 750 Welch Road # 210, Stanford, CA 94304, USA; SSallam@stanfordhealthcare.org (S.S.); NeShah@stanfordhealthcare.org (N.D.S.); WKwong@stanfordhealthcare.org (W.K.); dhkimmd@stanford.edu (D.K.)

**Keywords:** NAFLD, NASH, anti-diabetic medication, metformin, TZD

## Abstract

As a chronic disease encompassing a wide spectrum of liver-related histologic damage, nonalcoholic fatty liver disease (NAFLD) is becoming a global epidemic with significant impacts on all-cause morbidity and mortality. Insulin resistance and type 2 diabetes mellitus predispose individuals to NAFLD and related complications. Therefore, timely intervention with anti-diabetic medications may prevent and delay the development of NAFLD or have a therapeutic implication. The focus of this review is to evaluate the evidence supporting the efficacy of anti-diabetic medications in the treatment of NAFLD. While many of these anti-diabetic agents have shown to improve biochemical parameters, their effect on hepatic histology is limited. Among anti-diabetic medications, only thiazolidinediones and glucagon-like peptide-1 receptor agonists demonstrate significant improvement in hepatic histology.

## 1. Introduction

Nonalcoholic fatty liver disease (NAFLD) consists of hepatic irregularities ranging from intrahepatic accumulation of fat (steatosis or nonalcoholic fatty liver) to necrotic inflammation (nonalcoholic steatohepatitis; NASH). With the rise of global obesity, NAFLD is becoming the most common chronic liver disease with an estimated worldwide prevalence of 25.2% [1]. A total of 30% of patients with NAFLD continue to develop progressive fibrosis leading to NASH, thereby increasing their risk for hepatic decompensation and hepatocellular carcinoma (HCC) [2].

Although the pathogenesis of NAFLD is complex, the disease is closely linked to increased insulin resistance in hepatic and adipose tissues, as well as lower whole-body insulin sensitivity. Insulin resistance and obesity are shown to significantly contribute to NAFLD development and progression [3]. Studies have shown that decreased insulin responsiveness in adipose tissue can cause a flux of free fatty acid (FFA) dysfunction. Hepatic triglyceride accumulation, due to an imbalance of lipogenesis and FFA dysregulation, induces an inflammatory response and promotes liver injury, allowing for hepatic steatosis to occur [2,4]. Moreover, NAFLD is associated with a twofold increased risk of incident type 2 diabetes mellitus (T2DM) and metabolic syndrome [5,6]. Concurrent NAFLD is found in an estimated 70% of obese patients with T2DM [7].

NAFLD management is centered on lifestyle modifications, weight loss, and habitual physical activity. Weight loss promotes fat reduction and NAFLD remission. However, as NAFLD becomes more prevalent and where dietary changes and physical activity are difficult to maintain, there has been interest in evaluating a pharmaceutical approach. Insulin resistance and type 2 diabetes mellitus are strongly associated with NAFLD development and its fibrotic progression, which suggests that anti-diabetic medications may have therapeutic potential [8,9,10,11]. Various studies have focused on the use of anti-diabetic therapies with different mechanisms of action for NAFLD, including metformin, thiazolidinediones (TZDs), glucagon-like peptide 1 receptor (GLP-1r) agonists, dipeptidyl peptidase 4 (DPP-4) inhibitors, and sodium/glucose cotransporter 2 (SGLT2) inhibitors (Table 1). The focus of this review is to evaluate the evidence surrounding the efficacy of anti-diabetic medications in the treatment of NAFLD.

## 2. Metformin

Metformin, an oral biguanide, has been considered a first-line treatment for T2DM. The medication helps to lower blood glucose levels by reducing hepatic gluconeogenesis and gastrointestinal glucose absorption. Furthermore, metformin leads to weight loss which, in turn, improves insulin sensitivity. Several studies have investigated the impact of metformin to delay NAFLD progression [11,12]. In 2004, Nair et al. conducted an open label study in which 15 patients with histologically confirmed NAFLD were given continuous metformin therapy (20 mg/kg/day) for 1-year and compared pre-and post-treatment liver biopsies (Table 2). Results demonstrated transient improvement of liver function tests and minor histologic improvement in post-treatment liver biopsy specimens. This study showed no significant reduction in insulin levels was seen in patients after the first 3 months of treatment and liver function returned to baseline levels [12]. Similar results were also reported in another randomized placebo-controlled trial conducted by Haukeland et al., where 48 patients with biopsy-proven NAFLD received either metformin or a placebo for 6 months. Compared to the placebo group, patients treated with metformin had no significant improvement in liver histology, aminotransferase levels, or insulin sensitivity. However, the study did show a decrease in serum lipid and glucose levels. This suggests that metformin use could be beneficial in treating patients with NAFLD [13] (Table 2).

Various studies have examined the combination of metformin and lifestyle modifications versus lifestyle modifications alone in NAFLD management. Ugyn et al. reported a significant improvement in liver chemistries and insulin sensitivity in the metformin group. However, there were no significant histological improvements or changes in fibrosis in either group [14] (Table 2). Another randomized placebo controlled trial of 19 NAFLD patients that were grouped into either metformin plus dietary changes and exercise or diet and exercise alone, demonstrated no significant difference in histology in both treatment groups [15]. However, in a cohort of 250 patients with diabetes and concomitant end-stage liver disease or cirrhosis, patients who were continued on metformin had a longer median survival than those who discontinued metformin (11.8 vs. 5.6 years overall, *p* < 0.001) and a 57% reduction in all-cause mortality [16]. Although there is insufficient data suggesting metformin use for improvement in liver histopathology, it may improve all-cause mortality among those with NASH and associated end-stage liver disease [9,10,16].

## 3. Thiazolidinediones (TZDs)

TZDs improve insulin sensitivity by activating peroxisome proliferator-activated receptor γ (PPAR-γ) in the liver, muscle, and adipose tissues prim. In addition, TZDs increase levels of adiponectin, a protein hormone secreted by mature adipocytes. Adiponectin enhances insulin sensitivity by promoting glucose uptake and fatty acid oxidation. Increased fatty acid oxidation helps to decrease glucose production and results in reduced circulating fatty acid and triglyceride content in the liver [36]. TZDs can cause weight gain through a variety of mechanisms. TZDs lead to fat redistribution with a selection for an increase in subcutaneous fat, but a decrease for visceral fat. The net effect of weight gain is attributable to an increase in subcutaneous fat. However, despite weight gain, studies have shown that TZDs are able to decrease intrahepatic fat accumulation [37,38]. Among the various TZDs agents, rosiglitazone and pioglitazone have yielded promising results regarding its efficacy in the treatment of patients with NASH [36,39].

The Fatty Liver Improvement with Rosiglitazone Therapy (FLIRT) Trial evaluated 63 patients with biopsy-proven NASH who were given either rosiglitazone (8 mg/day) or a placebo for one year. Patients who took rosiglitazone experienced improvement in insulin sensitivity and normalization of alanine aminotransferase (ALT) levels four times greater than the placebo group. However, at 4-months post-treatment, the normalization was transient, and ALT returned to baseline levels. Other than a 30% significant decrease in hepatic steatosis, there was no significant histological improvement in the rosiglitazone group compared to the placebo group [17]. Based on these results, the FLIRT was extended by 2 years. However, no additional benefits were present with extended therapy [18].

In the Pioglitazone, Vitamin E, or Placebo for Nonalcoholic Steatohepatitis (PIVENS) trial, Sanyal et al. evaluated the effectiveness of pioglitazone and vitamin E therapy in the treatment ofNAFLD in patients without T2DM. Vitamin E has shown to reduce oxidative stress and associated hepatic mitochondrial dysfunction, a main trigger in the progression of simple steatosis towards steatohepatitis in patients with NAFLD. A total of 247 NASH patients without T2DM were randomly assigned into three groups and received pioglitazone (30 mg/day); vitamin E (800 IU/day); or placebo group for 96-weeks. Compared to the control group, both vitamin E and pioglitazone treatment groups experienced a significant decrease in hepatic steatosis and lobular inflammation. The PIVENS trial also reported that a greater percentage of patients in the pioglitazone group had NASH resolution compared to the placebo group (47% vs. 21%; *p* < 0.001) (Table 2). However, the pioglitazone and vitamin E group did not achieve significant improvement of fibrosis score [19]. Similar findings were also seen for pioglitazone therapy in NASH patients without diabetes [20].

Due to evidence of histological improvement, pioglitazone can be considered for treatment in biopsy-proven NASH according to American Association for the Study of Liver Diseases (AASLD) and European Association for the Study of Liver (EASL) guidelines [9,10]. The adverse effects, including weight gain, fluid retention, bone fractures, and the risk of cardiovascular events associated with TZDs remain a concern and may restrict usage [21].

## 4. Glucagon-Like Peptide 1 (GLP-1) Receptor (GLP-1r) Agonists

GLP-1 is a type of metabolic peptide hormone called incretin. It is secreted by enteroendocrine cells found in the distal ileum and colon in response to food consumption. GLP-1 plays an important role in glucose homeostasis by causing glucose dependent insulinotropic effects and inhibiting glucagon release [40]. In addition, GLP-1r agonists have substantial pleiotropic effects that help to promote weight loss and reverse hepatic steatosis [41,42]. Therefore, GLP-1r agonists, such as liraglutide, exenatide, and dulaglutide may have therapeutic potential to prevent the development of NAFLD and its progression [43].

In evaluating liraglutide’s effectiveness on liver function, Armstrong et al. conducted a meta-analysis of six randomized phase III trials in over 4400 diabetic patients for 26 weeks. Results showed dose-dependent reductions in liver ALT levels in treatment with liraglutide (1.8 mg/day) compared to placebo counterparts (*p* = 0.003) (Table 2). No statistically significant changes were seen in liver histology, but there was an observed trend towards improvement in hepatic steatosis. However, for both findings, the significance is greatly reduced after correcting for weight loss. Liraglutide treatment was examined as a therapeutic approach in patients with NASH and associated with improvement in some histologic parameters [22]. In a 2-year randomized, double-blind, placebo-controlled, phase 2 trial, patients with NASH treated with liraglutide had a higher percentage with histologic resolution (39% vs. 9%; *p* < 0.01) and lower fibrotic progression rates (9% vs. 36%; *p* < 0.05) [23].

Exenatide treatment has been studied in 217 diabetic patients as part of adjuvant therapy with metformin and/or sulfonylureas in a 3-year open label extension study. The study reported a 1% reduction in hemoglobin A1C (HgA1C) and 41% of patients with elevated baseline ALT achieved ALT normalization. Furthermore, in the patients with elevated baseline ALT, there was significant decrease in aminotransferase levels that trended with sustained weight loss. Adjunctive exenatide treatment has shown significant improvement in hepatic biomarkers, glucose control, and body weight [24]. An observational pilot study conducted by Garcia and colleagues reported ultrasonographic NAFLD improvement in 80% of diabetic patients treated with metformin combined with exenatide [25]. Shao et al. compared exenatide treatment plus insulin (glargine) versus intensive treatment with insulin (aspart and glargine) in obese patients with NAFLD and T2DM for 12 weeks. Compared to intensive insulin therapy, the exenatide treatment group experienced significant decreases in body weight, waist circumference, and liver enzyme activity. In addition, individuals treated with exenatide and glargine demonstrated a significantly higher reversal rate of fatty liver disease than the insulin-only treatment counterparts (93.3% vs. 66.7%; *p* < 0.01) [26].

A recent study evaluating the impact of dulaglutide on patients with concomitant NAFLD and T2DM reported significant reductions in aminotransferase levels and body weight. In addition there was improved HgA1C levels. Despite these results, further research is needed to assess the benefits and long-term outcomes of dulaglutide and other GLP-1r agonists [27]. 

## 5. Dipeptidyl Peptidase-4 (DPP-4) Inhibitors 

DPP-4 inhibitors or gliptins block DPP-4 enzyme prevents inactivation of incretins, including glucose-dependent insulinotropic polypeptide (GIP) and GLP-1. By prolonging incretin effect, DPP-4 inhibitors can stimulate insulin secretion, lower hepatic glucose output, and suppress glucagon release [44]. Compared to healthy subjects, hepatic expression of DPP-4 is significantly higher in patients with NAFLD. Therefore, DPP-4 inhibitors have been investigated as a novel therapeutic option for the management of NAFLD [45].

As one of the earliest commercially available DPP-4 inhibitors, sitagliptin has been extensively used to asses efficacy of DPP4 in patients with NAFLD. Improvement in serum aminotransferase levels was seen in an observational, single arm pilot study, in which 15 diabetic patients with NASH were given sitagliptin therapy for 1-year. Additionally, the study showed significant decreases in body mass index, NASH score, and hepatocyte ballooning. However, the effect of DPP-4 inhibitors on liver enzymes remains inconclusive [28].

In a 24 week randomized, double-blind, placebo-controlled study in NAFLD patients with impaired glucose tolerance or diabetes, Cui et al. reported no statistically significant histological improvement with sitagliptin compared to placebo [29] (Table 2). Similarly, another 24-week study with 12 biopsy-proven NASH patients also showed there was no significant differences in liver fibrosis and NASH activity score (NAS) when compared to placebo. The study did show trends towards decreasing adiponectin and HgA1C levels. However, the change in HgA1C was not clinically significant. Sitagliptin had no statistically significant differences associated withliver enzyme activity and histology in NASH patients [30].

Other DPP-4 inhibitor agents, alogliptin and vildagliptin, have also been explored for their clinical efficacy. In a 12-month non-randomized, multicenter, single-arm study of NAFLD patients with T2DM, alogliptin use was shown to limit NAFLD progression [31]. In addition, Macauley et al., in a 6-month randomized controlled trial in diabetic patients with good glycemic control, reported that vildagliptin use had a significant reduction of 27% in liver triglyceride levels, as well as improvement in ALT levels [32].

Overall, several studies have reported mixed results between DPP-4 inhibitors and liver enzyme activity. Additionally, there was no significant difference in liver histology in this patient population [28,29,30]. Therefore, due to insufficient available evidence, additional studies with larger sample sizes and an extended follow-up period are required to assess the effectiveness of DPP-4 inhibitors in the treatment of NAFLD/NASH with concurrent diabetes.

## 6. Sodium/Glucose Cotransporter-2 (SGLT2) Inhibitors

SGLT2 inhibitors or the gliflozins inhibit glucose reabsorption in the kidney by blocking SGLT2 protein found in proximal renal tubules. By blocking renal glucose reabsorption, the medication promotes urinary excretion of excess glucose and thereby lowering blood glucose levels independent of insulin secretion. Advantages of SGLT2 inhibitors include weight loss, low risk of hypoglycemia, and blood pressure reduction [46]. Previous animal models have demonstrated that SGLT2 inhibitors like ipragliflozin can have a preventative effect on steatohepatitis and fibrotic progression, irrespective of weight loss [47].

Seko et al. conducted a retrospective study that compared treatment between SGLT2 inhibitors and DPP-4 inhibitors in T2DM patients with biopsy proven NAFLD. Both drug groups had a significant reduction in aminotransferase levels. SGLT2 inhibitors experienced a significant decrease in body weight and improvement in glycemic control (Table 2) [33]. Further support is demonstrated in a retrospective study that evaluates the efficacy of SGLT2 inhibitors as a second line treatment for NAFLD patients with T2DM. Ohki et al. reported significant improvement of liver enzyme activity and fibrosis index with the addition of ipragliflozin [34]. Takase et al. conducted a prospective observational study to assess the effectiveness of ipragliflozin to reduce hepatic steatosis among 21 patients with T2DM after 16-weeks of treatment (Table 2). Although no correlation was seen in fatty liver changes, there were significant reductions in body weight, adipose tissue, and fat mass with ipragliflozin treatment [35].

Medications in this drug class, such as empagliflozin and canagliflozin, were found to have an increased likelihood of urinary tract and genital infections [48,49]. For the treatment of NAFLD, SGLT2 inhibitors are a relatively new drug class for the treatment of T2DM that may be beneficial therapeutic option after further studies evaluating the safety, efficacy, and tolerability in this sub-population.

## 7. Conclusions

As a chronic disease encompassing a wide spectrum of liver-related injuries, nonalcoholic fatty liver disease (NAFLD) is becoming a public health burden with significant impacts on all-cause morbidity and mortality. While many of these anti-diabetic drug agents have shown to improve biochemical parameters or all-cause mortality, their effect on liver histology is limited. Among anti-diabetic medications, only TZDs and GLP-1r agonists demonstrate significant histologic improvement (Table 1 and Table 2). In the management and resolution of NAFLD, current findings suggest that more research is required to assess the safety and efficacy of anti-hyperglycemic agents.

## Figures and Tables

**Table 1:** Comparative efficacy of anti-diabetic agents for the treatment of NAFLD.

Medications	Insulin Resistance	Body Weight	Body Fat	AST/ALT	Liver Histology
Biguanides (Metformin) [11,12,13,14,15,16]	Decreased	Decreased	Decreased	Inconclusive	Inconclusive
Thiazolidinediones [17,18,19,20,21]	Decreased	Increased	Increased	Decreased	Decreased
GLP-1r agonists [22,23,24,25,26,27]	Decreased	Decreased	Decreased	Decreased	Decreased
DPP-4 inhibitors [28,29,30,31,32]	Decreased	No Effect	No Effect	Decreased/No Effect	No Effect
SGLT2 inhibitors [33,34,35]	Decreased	Decreased	Decreased	Decreased	Inconclusive

**Table 2:** Anti-diabetic therapy for NAFLD and associated outcomes on liver histology and aminotransferases.

Study	Therapy	Outcomes
Nair et al. [12]	Metformin (20 mg/kg/day); 12 months	Histology minor Improvement Decreased ALT and AST
Haukeland et al. [13]	Metformin vs placebo; 6 months	Histology not improved Decreased ALT and AST
Ugyn et al. [14]	Metformin (1.7 g/day) + diet vs diet; 6 months	Histology not improved Decreased ALT and AST
FLIRT Trial (Ratziu et al.) [17,18]	Rosiglitazone (8 mg/day) vs placebo; 12 months	Histology not improved Decreased ALT levels
PIVENS Trial (Sanyal et al.) [19]	Pioglitazone (30 mg/day) vs Vitamin E (800 IU/day) vs placebo; 24 months	Histology improved in both Pioglitazone and Vitamin E groups
Armstrong et al. [23]	Liraglutide (1.8 mg/day) vs placebo; 26 weeks	Histology improved Decreased ALT
Klonoff et al. [24]	Exenatide + Metformin and/or sulfonylurea; 36 months	Histology improved Decreased ALT
Garcia et al. [25]	Exenatide + Metformin	Ultrasound NAFLD improvement in 80% of diabetic patients
Shao et al. [26]	Exenatide + Insulin Glargine vs insulin (glargine/aspart); 3 months	Fatty liver regression in exenatide + insulin glargine group
Yilmaz et al. [28]	Sitagliptin 100 mg daily; 12 months	Decreased ALT and AST Histology improved
Cui et al. [29]	Sitagliptin 100 mg daily vs placebo; 6 months	Histology not improved
Mashitani et al. [31]	Alogliptin; 12 months	Limits NAFLD progression Histology not improved
Macauley et al. [32]	Vildagliptin; 6 months	Decreased ALT levels Histology not improved
Seko et al. [33]	SGLT2 Inhibitor vs DPP4 Inhibitor; 6 months	Both had decrease in AST and ALT levels SGLT2 inhibitor had significant decrease in body weight and improved glycemic control
Ohki et al. [34]	Ipragliflozin added to DPP4 inhibitor or GLP-1r agonist	Decrease ALT Improved fibrosis index
Takase et al. [35]	Ipragliflozin in T2DM; 4 months	Reduction in body weight, adipose tissue, and fat mass No change in fatty liver index
